# LAG-3^+^ tumor-infiltrating lymphocytes ameliorates overall survival in triple-negative breast cancer patients

**DOI:** 10.3389/fonc.2022.986903

**Published:** 2023-01-24

**Authors:** Guoming Hu, Shimin Wang, Songxiang Wang, Qiannan Ding, Liming Huang

**Affiliations:** ^1^Department of General Surgery (Breast and Thyroid Surgery), Shaoxing People’s Hospital, Shaoxing Hospital, Zhejiang University School of Medicine, Shaoxing, Zhejiang, China; ^2^Key Laboratory of Cancer Prevention and Intervention, Ministry of Education, Hangzhou, Zhejiang, China; ^3^Shaoxing Key Laboratory of Functional Molecular Imaging of Tumor and Interventional Diagnosis and Treatment, Shaoxing, Zhejiang, China; ^4^Department of Nephrology, Shaoxing People’s Hospital, Shaoxing Hospital, Zhejiang University School of Medicine, Shaoxing, Zhejiang, China; ^5^Medical Research Center Shaoxing People’s Hospital, Shaoxing Hospital, Zhejiang University School of Medicine, Shaoxing, Zhejiang, China

**Keywords:** LAG-3+tumor-infiltrating lymphocytes, tumor microenvironment, triple-negative breast cancer, favorable prognosis, meta-analysis

## Abstract

**Purpose:**

Immune checkpoint molecule lymphocyte-activating gene-3 (LAG-3), which is expressed on active lymphocytes, has proven to be associated with immunosuppression and cancer progression in a variety of solid tumors. However, the role of LAG-3^+^ lymphocytes in human breast cancer (BC) is still not conclusive. We therefore performed a meta-analysis to clarify the role of these cells in prognosis prediction for BC.

**Methods:**

We searched PubMed, Embase, and EBSCO to identify the studies evaluating the association of LAG-3^+^ lymphocyte infiltration and overall survival (OS) and/or disease-free survival (DFS) in BC patients, then combined extracted data with STATA 12.0.

**Results:**

Eight published studies involving 5,859 BC patients were incorporated into this meta-analysis. We noted that a high number of LAG-3^+^ tumor-infiltrating lymphocytes were not appreciably associated with OS and DFS in BC patients. Strikingly, in stratified analyses based on the molecular type of BC, LAG-3^+^ lymphocyte infiltration was remarkably associated with better OS rather than DFS in triple-negative breast cancer (TNBC), whereas it significantly influenced neither OS nor DFS in Her2-positive BC. However, an increased density of these lymphocytes indicated a trend for better OS in Her2-positive BC. In addition, we found that LAG-3^+^ lymphocyte infiltration was also remarkably associated with prolonged OS in Her2-positive BC patients when they were measured by immunohistochemistry (IHC). In addition, an elevated number of these lymphocytes did not correlate with pathological complete response rate or clinicopathological features including lymph node metastasis.

**Conclusion:**

The infiltration of LAG-3^+^ lymphocytes ameliorates OS in TNBC and Her2-positive BC, implicating that it is a valuable prognostic biomarker, and applications of anti-LAG-3 antagonists may possibly be not a promising therapeutic strategy for human BC especially for TNBC.

## Introduction

Human breast cancer is one of the most common fatal malignancies in women worldwide. The tumor microenvironment (TME) links closely with the initiation, promotion, and progression of breast cancer (BC) *via* diverse mechanisms such as inducing immune suppression and angiogenesis (1). Immune checkpoint molecules such as programmed cell death 1 (PD-1) and its ligand (PD-L1), cytotoxic T lymphocyte antigen 4 (CTLA-4), have proven to be associated with the formation of the immunosuppressive microenvironment and immune evasion in multiple cancers (2). Currently, immune checkpoint inhibitors such as anti PD-1/PD-L1 antagonists have been utilized in clinical practice, yielding impressive results especially in lung carcinoma (3). However, these inhibitors showed very limited benefit for patients with other solid tumors including BC (4).

Lymphocyte-activating gene-3 (LAG-3) (also known as CD233) is a type I transmembrane protein with structural similarities to CD4. Accumulating evidence has demonstrated that LAG-3 is an inhibitory coreceptor and a new immune checkpoint molecule, which exerts pivotal roles in anti-infection immunity, autoimmunity, and tumor immunity (5). LAG-3 expressed on activated T lymphocytes correlated with T-cell exhaustion, thereby leading to dysfunction of antitumor immunity mediated by effector T cells (6). Recently, the infiltration of LAG-3^+^lymphocytes has been reported to be associated with tumor progression and poor prognosis in a variety of human cancers such as colorectal cancer (7), renal cell carcinoma (8), head and neck squamous cell carcinoma (HNSCC) (9), and non-small cell lung cancer (10) However, the relationship between LAG-3^+^ tumor-infiltrating lymphocytes and prognosis in human BC is still not conclusive (11, 12). Hence, it needs further evaluation to clarify the potential of these cells as an effective prognostic biomarker for BC patients.

Herein, we performed this meta-analysis to quantitatively summarize the relationship between LAG-3^+^ lymphocyte infiltration and clinical outcomes such as overall survival (OS) and disease-free survival (DFS) in BC patients and thereby provided more evidence on the clinical value of these lymphocytes as a prognostic biomarker and LAG-3-targeted therapeutic strategy for BC.

## Materials and methods

### Search strategy

We searched PubMed, Embase, and EBSCO for studies to test the density of LAG-3^+^ tumor-infiltrating lymphocytes and survival in BC patients from 1980 to 31/05/2022. The keywords adopted for search were (LAG-3 [All Fields] OR CD233 [All Fields]) AND (breast [All Fields] OR mammary [All Fields]) AND (neoplasms [All Fields] OR tumor [All Fields] OR cancer [All Fields] OR carcinoma [All Fields]). A total of 44, 72, and 108 entries were identified in PubMed, Embase, and EBSCO, respectively.

### Inclusion and exclusion criteria

Inclusion criteria of the meta-analysis were as follows: studies must have (1) been published as original articles in English; (2) investigated BC patients; and (3) provided hazard ratios (HRs) with 95% confidence interval (CI) or Kaplan–Meier curves of high and low density of LAG-3^+^ lymphocytes with OS and/or DFS.

We excluded studies that were not published as research articles or full texts including commentaries, case report, letters to editors and conference abstracts, studies that did not provide sufficient data to estimate hazard ratios (HRs), and studies that detected LAG-3^+^ lymphocytes in peripheral blood or metastatic sites.

### Endpoints

In this meta-analysis, we recorded OS as the primary endpoint, whereas DFS was considered as the second endpoint. Individual studies defined cutoffs of LAG-3^+^ lymphocytes and classified patients into high and low groups.

### OS and DFS definition

OS was defined as the time from the date of the first curative operation to the date of the last follow-up, or death from any cause, whereas DFS was the time from the date of the first curative surgery to the date of the first loco-regional or systemic relapse, or death without any type of relapse.

### Data extraction

Two authors (GM.H. and SM.W.) independently reviewed and extracted information including first author’s name, publication year, number of patients, neoadjuvant therapy (NAT) received or not, time of follow-up, and cutoff value determining a high level of LAG-3^+^ lymphocytes. OS, DFS, pathological complete response (pCR) rate, and clinicopathological data including lymph node metastasis and tumor differentiation were extracted from the text or tables.

### Quality assessment

Two independent authors appraised the quality of included individual studies with the Newcastle–Ottawa Scale (NOS) (13) and achieved consensus for each item with the help of a third author. A score of 6 or above was considered as high quality.

### Statistical analysis

We combined relevant data into hazard ratios (HRs) for OS, DFS, and odds ratios (ORs) for pCR rates of NAT and clinicopathological features such as lymph node metastasis with STATA 12.0 according to the random-effect model if statistical heterogeneity was remarkable (14); otherwise, the fixed-effect model was adopted (15, 16). In addition, we also applied sensitivity analysis as well as Begg’s funnel plot and Egger’s test to investigate the influence of each study on the pooled result and potential publication bias, respectively (17). All *P* values were two-sided, and those below 0.05 were considered as statistical significance.

## Results

### Search results and description of studies

There were 224 records retrieved, and the results are shown in **Figure S1**. We ultimately included eight studies with 5,859 breast cancer patients for the evaluation of LAG-3^+^ tumor-infiltrating lymphocytes (11, 12, 18–23) and then appraised all these studies with the Newcastle–Ottawa Scale (NOS). Characteristics of included studies which were in the light of the inclusion criteria and appropriate for data mergence are shown in **Table 1** and **Table S1**.

**Table 1:** Main characteristics of the included studies.

Study	Year	Tumor type	No. of patients	Median age (range) (year)	Cutoffs	Method for detection	LAG-3^+^ tumor-infiltrating lymphocyte density: H/L	Treatment	Tumor stage (TNM)	Median follow-up date (months)	Survival	Quality score (NOS)
Bagbudar et al. (11)	2022	Invasive BC	238	51 (27, 88)	1%/0.3 mm2	IHC	179/59	Surgery, without NAT	I–III	79 (9, 130)	OS, DFS	8
Stovgaard et al. (18)	2022	TNBC	488	≥50:72.7%; <50:27.3%	>10/0.3 mm2	IHC	300/188	Surgery, without NAT	I–III	NR	OS, DFS	8
Cabioglu et al. (19)	2021	TNBC	61	49 (24, 76)	>1%/0.3 mm2	IHC	16/45	NAT and surgery	II–III	47 (12, 204)	OS, DFS	7
Sarradin et al. (20)	2021	TNBC	66	52 (24, 71)	>0/0.3 mm2	IHC	51/15	NAT and surgery	II–III	35.4 (26.5, 44.4)	OS	7
Wang et al. (21)	2018	TNBC	148	NR	1%/0.3 mm2	IHC	33/115	NAT and surgery	II–III	NR	DFS	8
Fang et al. (22)	2020	Invasive BC (all)	1,764	(42, 80)	NR	NGS (206486 at)	NR	Surgery, without NAT	NR	NR	OS, DFS	7
		TNBC	153	NR								
		Her2-positive BC	73	NR								
Burugu et al. (23)	2017	Invasive BC (all)	2,917	NR	>1/0.3 mm2	IHC	326/2,591	Surgery, without NAT	NR	NR	OS, DFS	8
		TNBC	276	NR			90/186					
		Her2-positive BC	200	NR			41/159					
Asano et al. (12)	2022	Invasive BC (all)	177	≤56: 49.2%; >56: 50.8%	NR	IHC	47/130	NAT and surgery	NR	40.8 (7.2, 72)	OS, DFS	7

BC, breast cancer; TNBC, triple-negative breast cancer; OS, overall survival; DFS, disease-free survival; NAT, neoadjuvant therapy; IHC, immunohistochemistry; H, high; L, low; NGS, “next-generation” sequencing technology.

### Meta-analyses

#### OS

**Figure 1:**
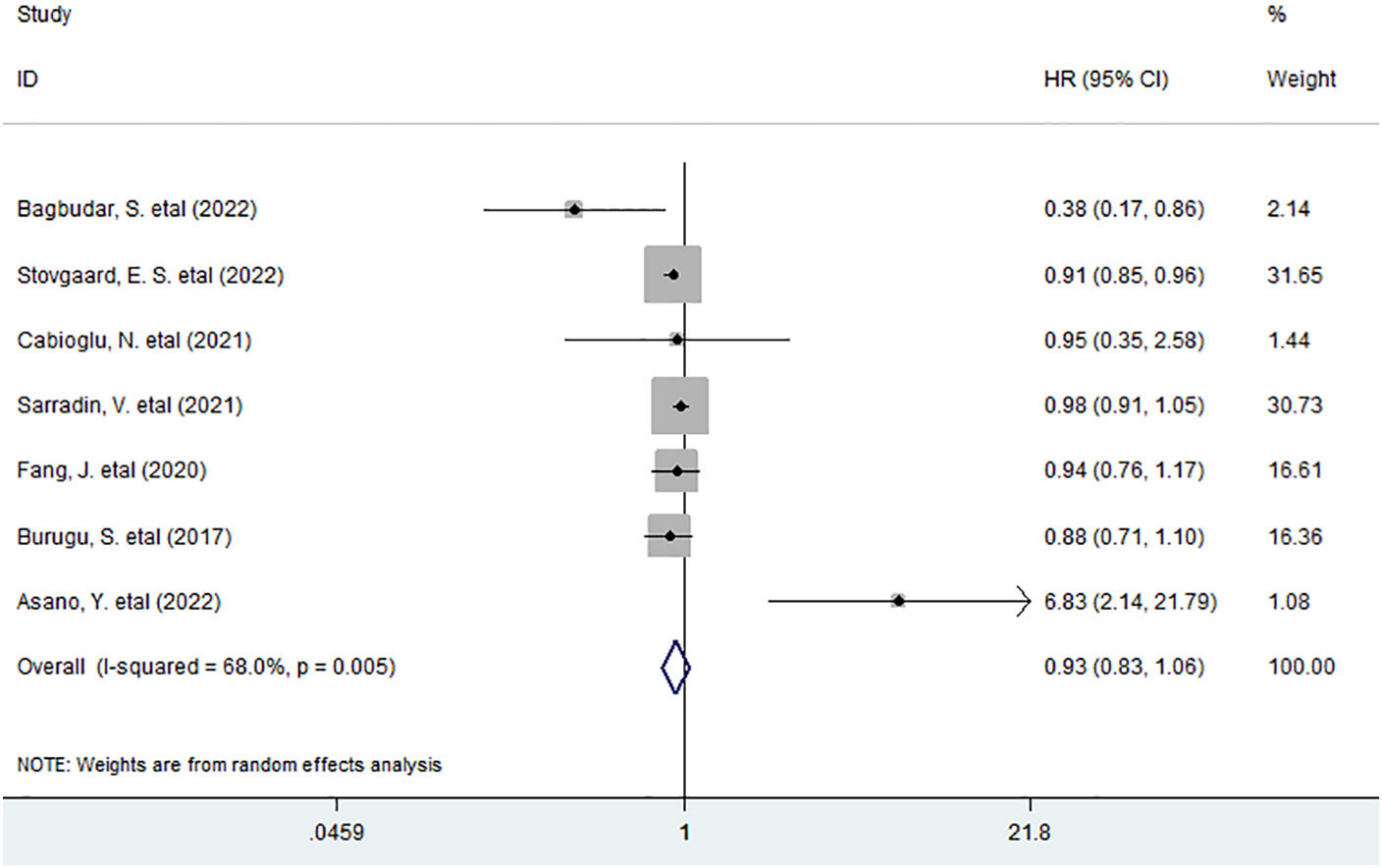
Forest plots describing HR of the association between LAG-3^+^ tumor-infiltrating lymphocytes and OS in breast cancer patients.

As for the association between high density of LAG-3^+^ tumor-infiltrating lymphocytes and OS in BC, we included seven studies with 5,711 patients for data combination. The pooled data indicated that an elevated density of LAG-3^+^ lymphocytes within tumor had no considerable prognostic effect on OS (HR = 0.93, 95% CI 0.83 to 1.06, *P* = 0.276) in all BC patients (**Figure 1**).

**Figure 2:**
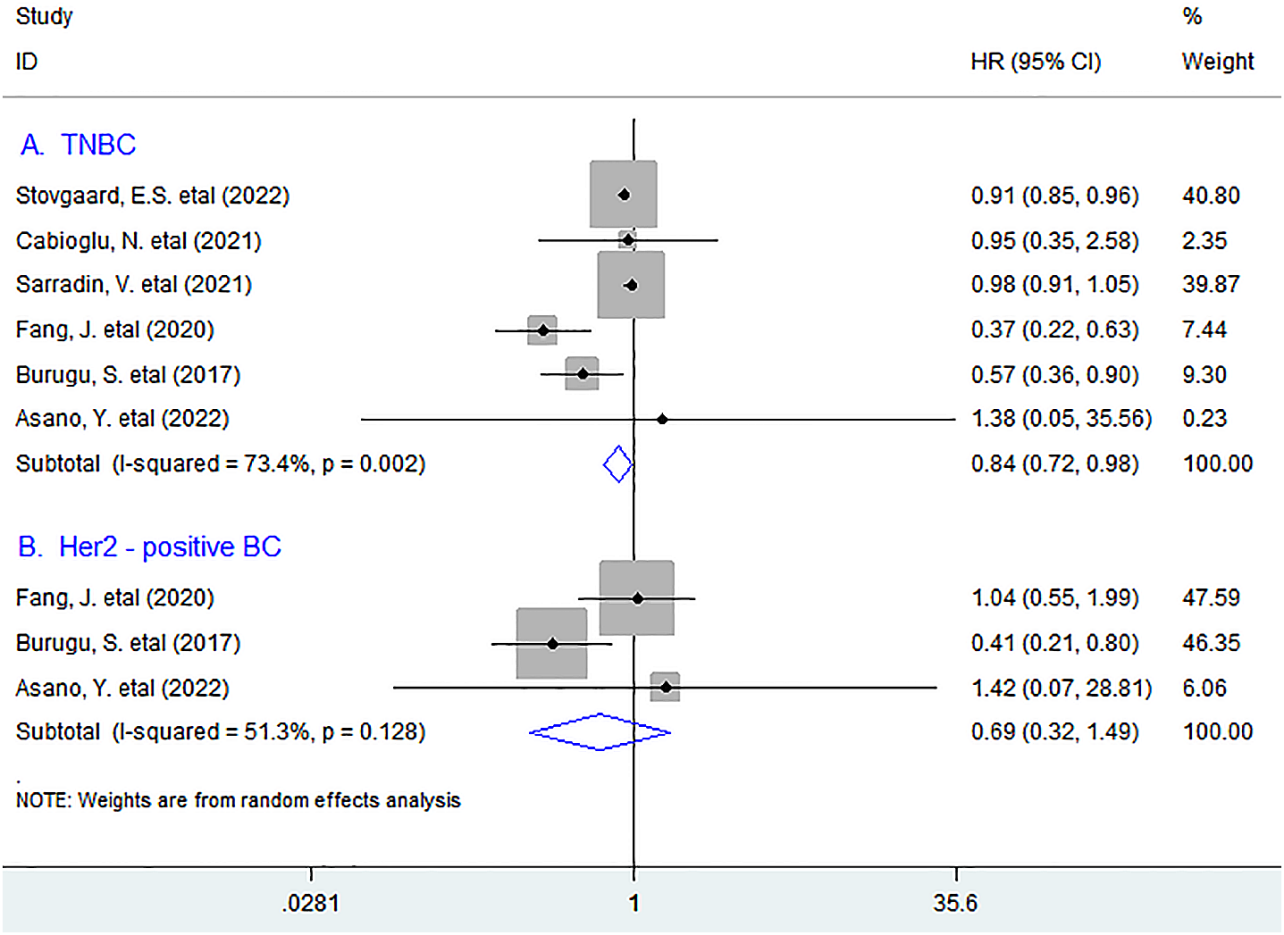
Stratified analyses describing HRs of the association between LAG-3^+^ tumor-infiltrating lymphocytes and OS in TNBC and Her2-positive breast cancer. TNBC, triple-negative breast cancer.

In stratified analyses based on molecular type of BC such as triple-negative BC (TNBC), Her2-positive BC, and luminal BC, as shown in **Figure 2**, six studies with 1,105 TNBC patients were pooled into the subgroup analysis. The results showed that LAG-3^+^ lymphocyte infiltration was appreciably associated with improved OS in TNBC patients (HR = 0.84, 95% CI 0.72 to 0.98, *P* = 0.031), whereas increased density of LAG-3^+^ tumor-infiltrating lymphocytes indicated a trend for better OS but was not significant in Her2-positive BC patients (HR = 0.69, 95% CI 0.32 to 1.49, *P* = 0.345).

**Figure 3:**
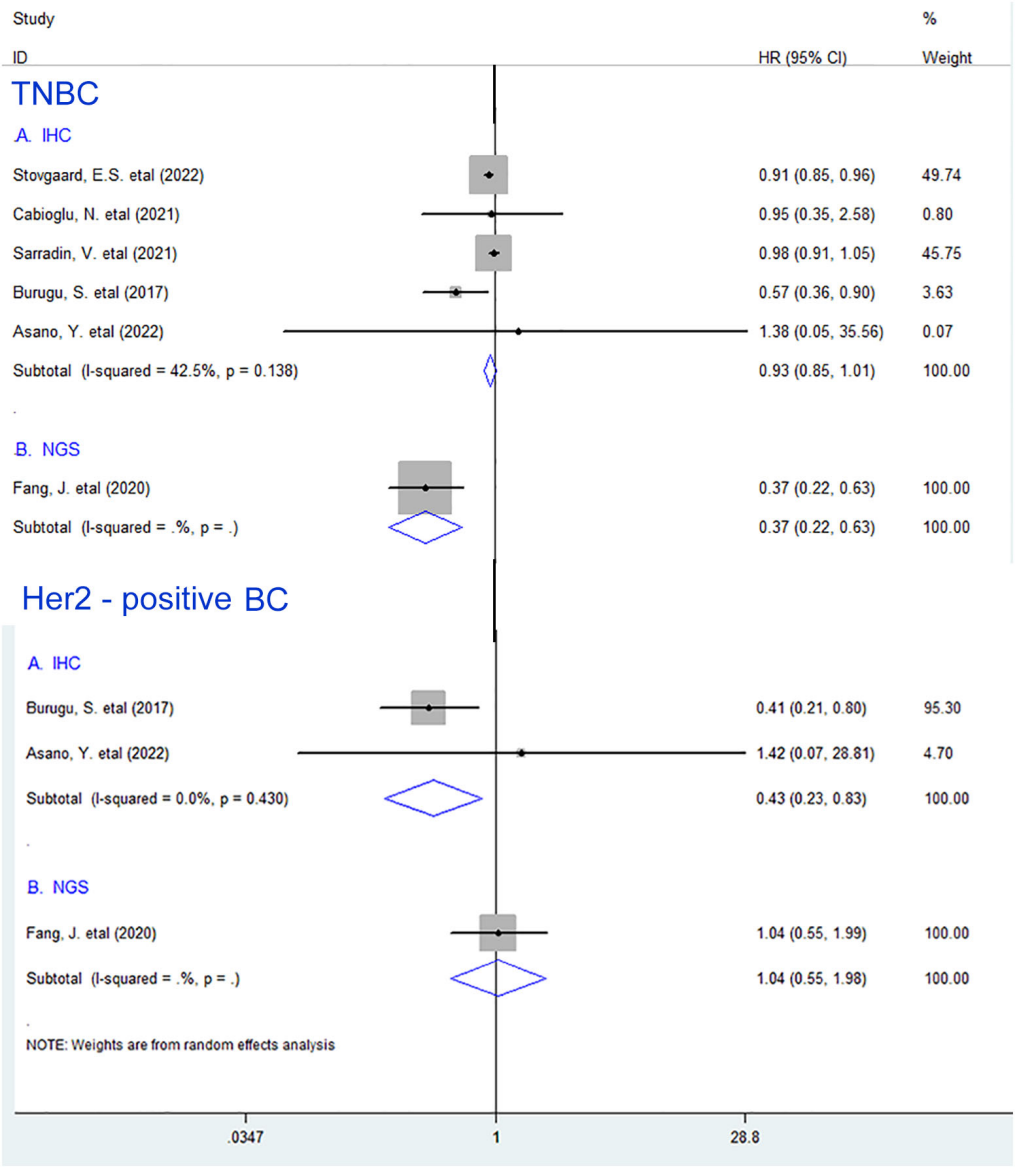
Stratified analyses describing HRs of the association between LAG-3^+^ tumor-infiltrating lymphocytes and OS in TNBC and Her2-positive breast cancer according to the detection methods. TNBC, triple-negative breast cancer; IHC, immunohistochemistry; NGS, “Next-generation” sequencing technology.

In addition, we further performed subgroup analyses according to the detection methods for LAG-3^+^ lymphocytes in TNBC and Her2-positive BC and noted that LAG-3^+^ lymphocyte infiltration was remarkably associated with prolonged OS in Her2-positive BC (HR = 0.43, 95% CI 0.23 to 0.83, *P* = 0.012), whereas it only showed a trend for improved OS in TNBC (HR = 0.93, 95% CI 0.85 to 1.01, *P* = 0.094) patients when such subset was measured by immunohistochemistry (IHC) (**Figure 3**).

#### DFS

**Figure 4:**
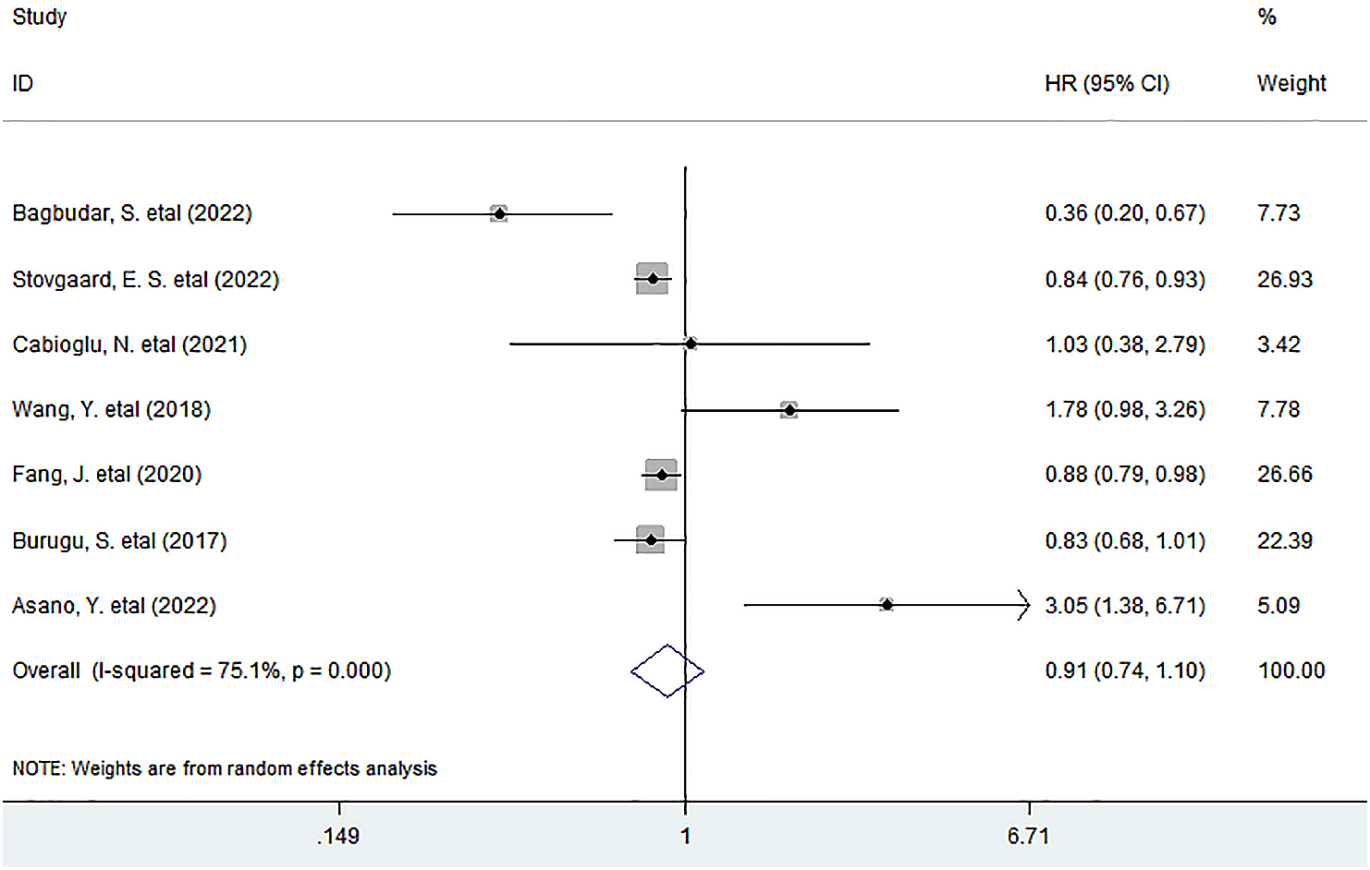
Forest plots describing HR of the association between LAG-3^+^ tumor-infiltrating lymphocytes and DFS in breast cancer patients.

Seven studies with the IHC method involving 5,793 patients investigated the association between LAG-3^+^ tumor-infiltrating lymphocytes and DFS, and the meta-analysis exhibited that LAG-3^+^ lymphocyte infiltration was not associated with DFS in all BC patients (HR = 0.91, 95% CI 0.74 to 1.11, *P* = 0.323) (**Figure 4**).

**Figure 5:**
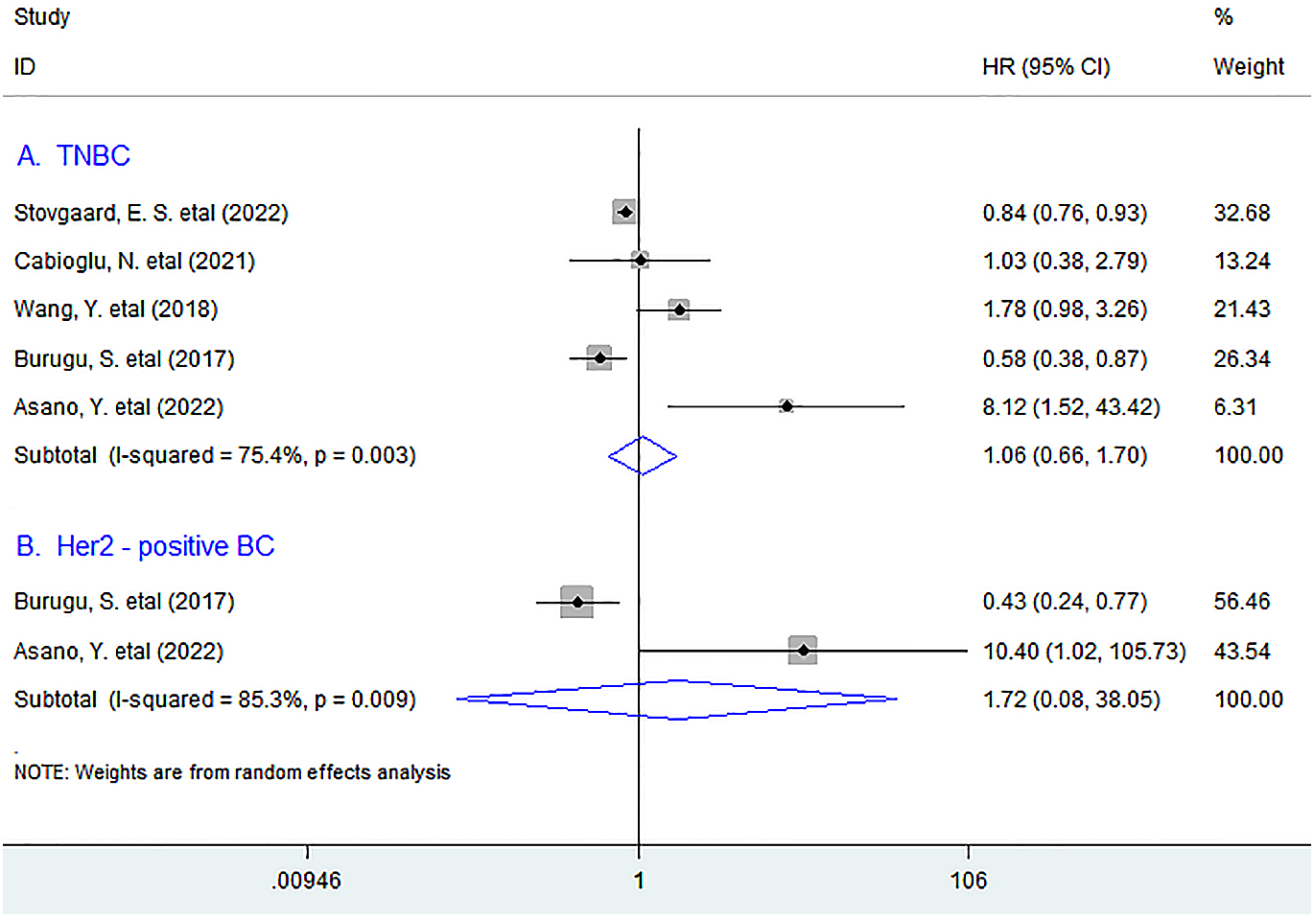
Stratified analyses describing HRs of the association between the LAG-3^+^ tumor-infiltrating lymphocytes and DFS in TNBC and Her2-positive breast cancer. TNBC, triple-negative breast cancer.

In stratified analyses, we found that there was no significant association between the infiltration of LAG-3^+^lymphocytes and DFS in TNBC (HR = 1.06, 95% CI 0.66 to 1.70, *P* = 0.802). A similar result was observed between LAG-3^+^ lymphocyte infiltration and DFS in Her2-positive BC patients (HR = 1.72, 95% CI 0.08 to 38.05, *P* = 0.731) (**Figure 5**).

We next investigated whether LAG-3^+^ tumor-infiltrating lymphocytes correlated with the pCR rate of NAT or clinicopathological features such as lymph node metastasis and primary tumor stage of BC. We observed that LAG-3^+^ lymphocyte infiltration was neither noticeably associated with pCR rate (OR = 1.06, 95% CI 0.18 to 6.21, *P* = 0.945) in TNBC nor with primary tumor stage (T1/T2) (OR = 1.02, 95% CI 0.54 to 1.91, *P* = 0.957), lymph node metastasis (OR = 1.19, 95% CI 0.89 to 1.58, *P* = 0.243), or tumor differentiation (OR = 0.68, 95% CI 0.20 to 2.38, *P* = 0.552) of all BC patients (**Figure S2**).

### Sensitivity analysis

Sensitivity analysis demonstrated that each included research had no impact on the overall result for OS or DFS. (**Figure S3**).

### Publication bias

Funnel plot and Egger’s test indicated that no significant publication bias existed between high density of LAG-3^+^ tumor-infiltrating lymphocytes and OS (*P* =0.764) or DFS (*P* =0.548) in BC patients.

## Discussion

In preclinical studies, LAG-3 expressing on tumor-infiltrating T cells could confer them immunosuppressive property and promote tumor progression in the TME, thereby predicting worse prognosis. However, in this study, we unexpectedly found that there was no significant association between high density of LAG-3^+^ tumor-infiltrating lymphocytes and survival including OS and DFS in BC patients. Strikingly, in subgroup analyses based on molecular type of BC, LAG-3^+^ lymphocyte infiltration was appreciably associated with better OS rather than DFS in TNBC, whereas it did not affect OS or DFS significantly in Her2-positive BC. However, LAG-3^+^ lymphocyte infiltration was remarkably associated with prolonged OS in Her2-positive BC rather than in TNBC patients when they were measured by IHC. In addition, an elevated number of these lymphocytes did not correlate with pCR rate after NAT or clinicopathological features. These findings suggested that LAG-3^+^ tumor-infiltrating lymphocytes might not promote but impede tumor progression in human BC.

TNBC is usually characterized by an aggressive phenotype, associated with poor prognosis. Current approaches are limited to chemotherapy due to the lack of specific therapeutic targets (24). Previous studies have shown that patients with TNBC would be more likely to achieve pCR after NAT, owing to an abundance of TILs in TME, and high expression of LAG-3 in tumor tissue correlated with more tumor-infiltrating CD8^+^T cells in TNBC (21), suggesting that an elevated level of LAG-3 before treatment is associated with a high pCR rate in TNBC. However, in this study, we noted that LAG-3^+^ lymphocyte infiltration did not notably improve the pCR rate in TNBC after NAT, which might attribute to the heterogeneous composition of the TME. In addition, detection methods for LAG-3^+^ lymphocytes appeared to influence their prognostic effect in TNBC and Her2-positive BC, and further investigation with much more studies should be performed to verify these results.

Prior data have suggested that the presence of an active immune system exerted key roles in preventing long-term recurrence in early-stage disease, contributing to the maintenance of cancer cells in a dormant status, although the underlying mechanism has not been clarified (25). In addition, multiple studies have demonstrated that the infiltration of memory T lymphocytes and CD8^+^ T cells was predictive of better prognosis in human BC (26, 27); thus, the infiltration of LAG-3^+^ lymphocytes that were positively correlated with the high density of CD8^+^T cells in the TME of TNBC (21) would reflect an active host antitumor immunity, thereby improving survival.

There was a paradoxical phenomenon that an elevated expression of immune checkpoint molecules such as CTLA-4 and PD-L1 and improved survival of cancer patients (28, 29), as these molecules could induce an immunosuppressive microenvironment and promote immune evasion and tumor progression. However, current data also suggested that the presence of immune checkpoint molecules expressing TILs might in fact indicate that there was a developing cancer-immune interaction, which was described as an inflamed tumor such as TNBC (30) and usually implicates better prognosis.

There were some limitations in this meta-analysis. The cutoff values were inconsistent between included studies; in addition, morphometric analyses for LAG-3^+^ lymphocytes used in individual included studies were not consistent, which might cause statistical heterogeneity. Finally, there was only one or two studies included in some subgroup analyses.

In conclusion, an elevated density of LAG-3^+^ tumor-infiltrating lymphocytes ameliorates OS in TNBC, implicating that it is a valuable prognostic biomarker, and applications of anti-LAG-3 antagonists may possibly not be a promising therapeutic strategy for human BC especially for TNBC.

## Data availability statement

The original contributions presented in the study are included in the article/**Supplementary Material**. Further inquiries can be directed to the corresponding authors.

## Author contributions

GMH conceived of the study, participated in its design, extracted data, performed the statistical analysis, and drafted the manuscript; SMW participated in data extraction; SXW and QND participated in data curation. LMH participated in the design of the study. All authors read and approved the final manuscript.
