# Supplementary material for: LAG-3+ tumor-infiltrating lymphocytes ameliorates overall survival in triple-negative breast cancer patients
Source: Front Oncol. 2023 Jan 24;12:986903. doi: 10.3389/fonc.2022.986903 (PMC9904386; doi:10.3389/fonc.2022.986903)
Supplement: Supplementary file 1 [file DataSheet_1.docx]

**LAG-3^+^Tumor-infiltrating Lymphocytes Ameliorates Overall Survival in Triple-negative Breast Cancer Patients**

Guoming Hu ^1, 2,^ *, Shimin Wang ^3^, Songxiang Wang ^1^,Qiannan Ding ^4^, Liming Huang ^1^

^1^ *Department of General Surgery (Breast and Thyroid Surgery), Shaoxing People’s Hospital; Shaoxing Hospital, Zhejiang University School of Medicine; 312000, Zhejiang, China (Key Laboratory of Cancer Prevention and Intervention, Ministry of Education).*

*^2^ Shaoxing Key Laboratory of Functional Molecular Imaging of Tumor and Interventional Diagnosis and Treatment.*

*^3^ Department of Nephrology, Shaoxing People’s Hospital; Shaoxing Hospital, Zhejiang University School of Medicine; 312000, Zhejiang, China.*

*^4^ Medical Research Center Shaoxing People’s Hospital; Shaoxing Hospital, Zhejiang University School of Medicine; 312000, Zhejiang, China.*

**
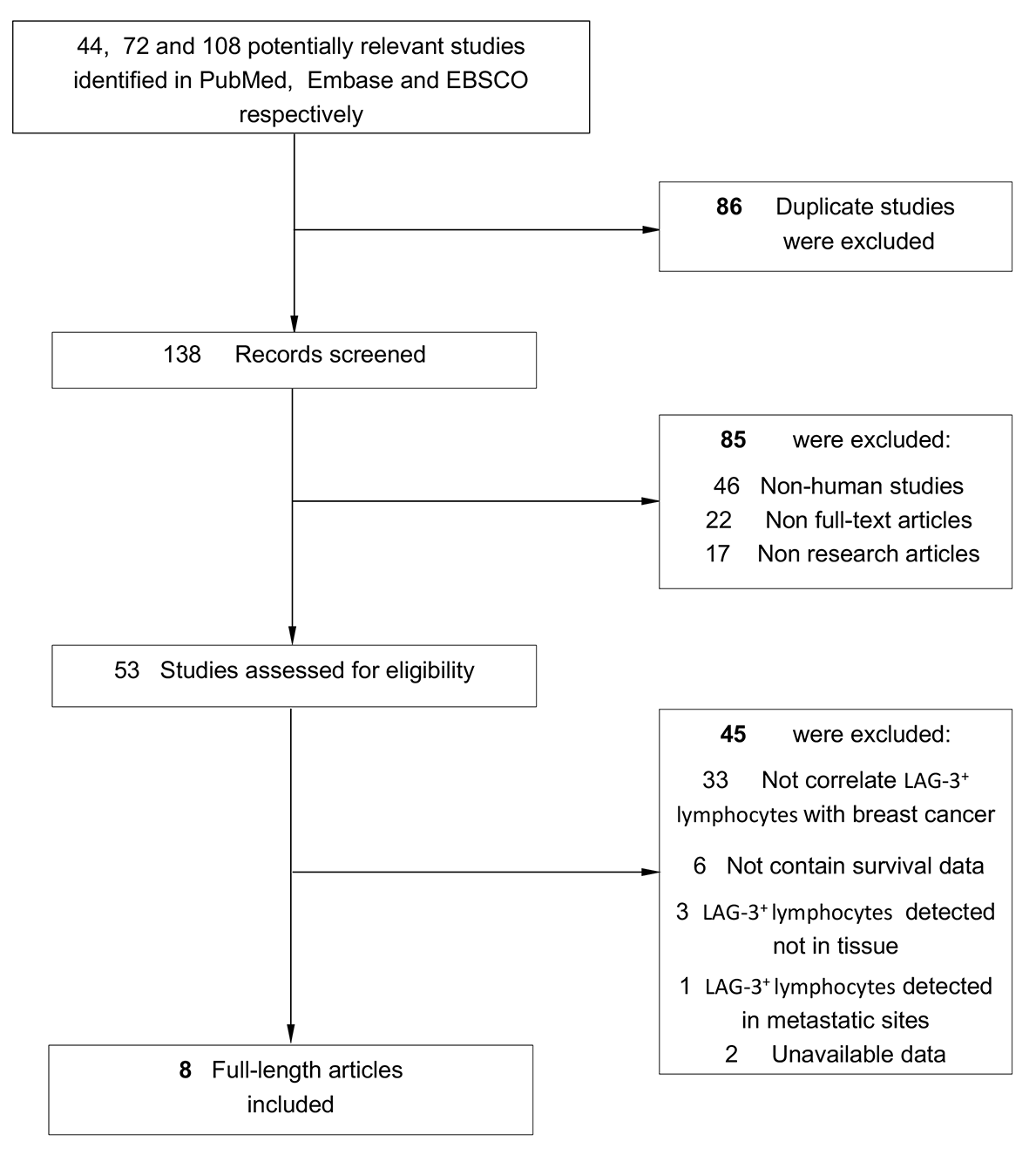
**

**Fig. S1.** Flow chart diagram of study selection.


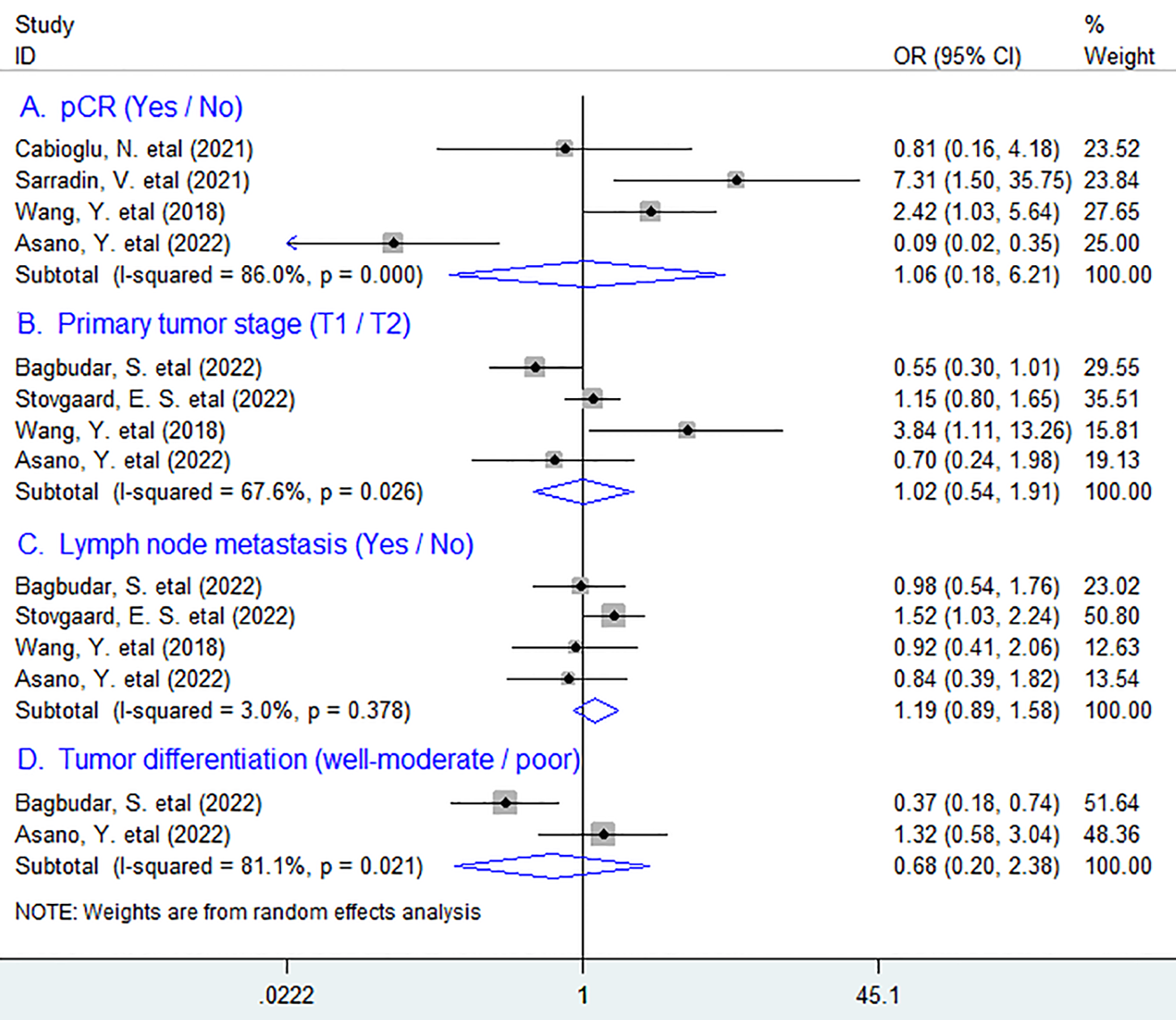


**Fig. S2.** Forest plots indicating ORs of the association between LAG-3^+^tumor-infiltrating lymphocytes and pCR rate of NAT (**A**) and clinicopathological features including primary tumor stage (**B**), lymph node metastasis (**C**), tumor differentiation (**D**). pCR: pathological complete response; NAT: neoadjuvant therapy

**
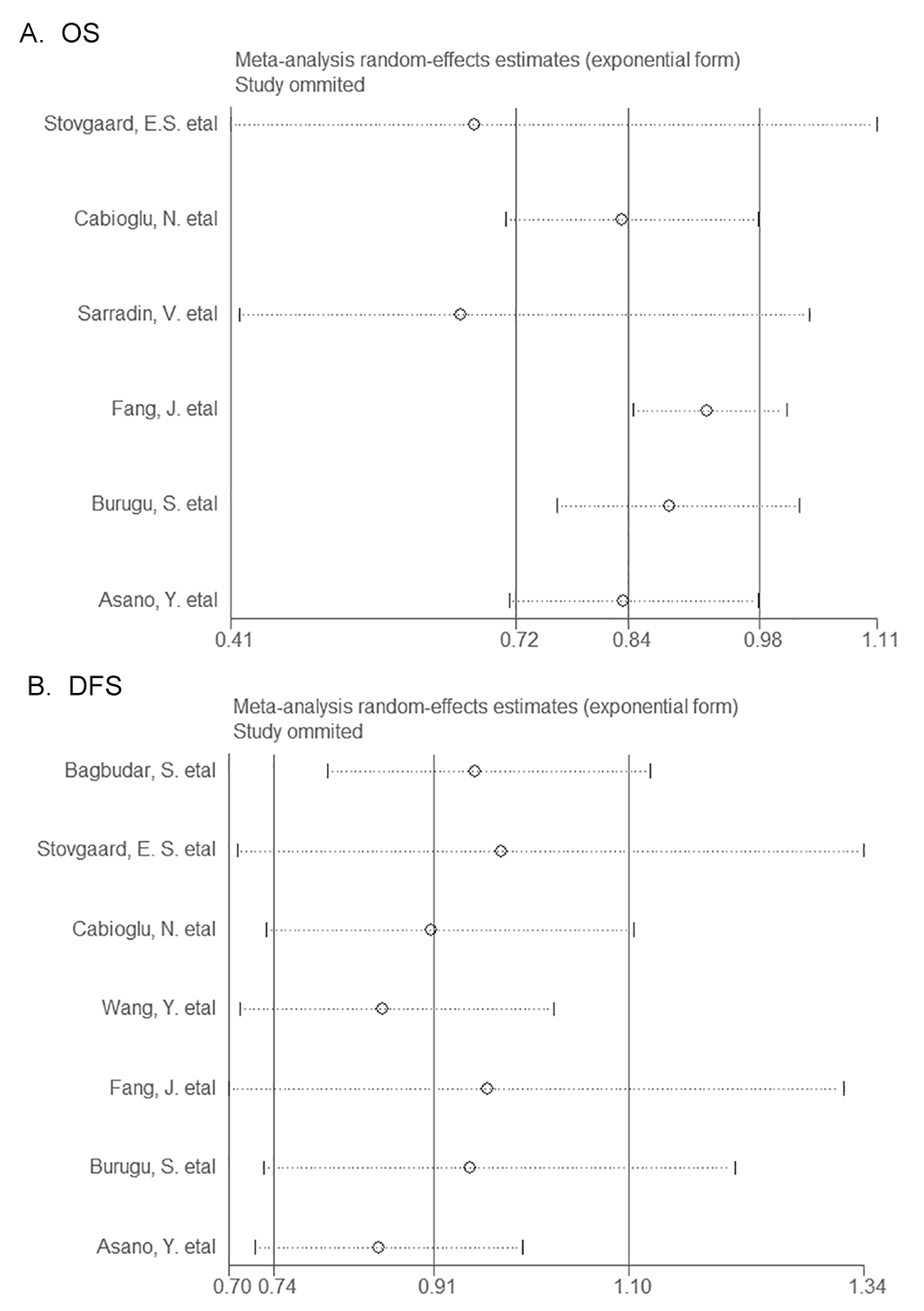
**

**Fig. S3.** Plots describing the influence of individual studies on the overall HRs for OS (**A**) and DFS (**B**) in human breast cancer. OS: overall survival; DFS: disease-free survival.

**Table S1.** Characteristics of the included studies for OR analysis of pCR rate and clinicopathological features.

| **Study** | **Year** | **Tumor type** | **No. of Patients** | **LAG-3^+^tumor-infiltrating lymphocyte density: H / L** | **pCR**  **(Yes/No)** | **Lymph node metastasis**  **(P / N)** | **Primary tumor stage (T1/T2)** | **Tumor differentiation (well-moderate / poor)** |
| --- | --- | --- | --- | --- | --- | --- | --- | --- |
| Bagbudar, S. etal [11] | 2022 | Invasive BC | 238 | 179/59 | NR | H:(96/83); L:(32/27) | H:(57/122); L:(27/32) | H:(25/154); L:(18/41) |
| Stovgaard, E. S. etal [18] | 2022 | TNBC | 488 | 300/188 | NR | H:(120/175); L:(57/126) | H:(145/155); L:(84/103) | NR |
| Cabioglu, N. etal [19] | 2021 | TNBC | 61 | 16/45 | H:(2/9); L:(14/51) | NR | NR | NR |
| Sarradin, V. etal [20] | 2021 | TNBC | 66 | 51/15 | H:(27/24); L:(2/13) | NR | NR | NR |
| Wang, Y. etal [21] | 2018 | TNBC | 148 | 33/115 | H:(12/21); L:(22/93) | H:(12/21); L:(44/71) | H:(6/19); L:(6/73) | NR |
| Asano, Y. etal [12] | 2022 | Invasive BC (all) | 177 | 47/130 | NR | H:(35/12); L:(101/29) | H:(5/42); L:(19/111) | H:(38/9); L:(99/31) |
|  |  | TNBC | 61 | 22/39 | H:(3/19); L:(25/14) | NR | NR | NR |
|  |  | Her2-positive BC | 36 | 15/21 | NR | NR | NR | NR |

TNBC: triple-negative breast cancer; pCR: pathological complete response; P: positive; N: Negative; T: primary tumor; NR: not reported.
